# Adult pancreatic hemangioma in pregnancy – concerns and considerations of a rare case

**DOI:** 10.1186/s12893-015-0106-1

**Published:** 2015-10-30

**Authors:** Jon Arne Søreide, Ole Jakob Greve, Einar Gudlaugsson

**Affiliations:** Department of Gastrointestinal Surgery, Stavanger University Hospital, N-4068 Stavanger, Norway; Department of Radiology, Stavanger University Hospital, Stavanger, Norway; Department of Pathology, Stavanger University Hospital, Stavanger, Norway; Department of Clinical Medicine, University of Bergen, Bergen, Norway

**Keywords:** Cystic lesion, Hemangioma, Pancreas, Pregnancy

## Abstract

**Background:**

Pancreatic tumors in pregnancy are rare but clinically challenging. Careful diagnostic workup, including appropriate imaging examinations, should be performed to evaluate surgery indications and timing . In the present case a diagnosis of an adult pancreatic hemangioma was made. We were not able to identify a similar case in the very sparse literature on this rare disease.

**Case presentation:**

A 30-year-old woman at 12 weeks of gestation was diagnosed with a large pancreatic tumor having a cystic pattern based on imaging. Although the preoperative diagnosis was uncertain, patient preference and clinical symptoms and signs suggested surgery. Open distal pancreatic resection including splenectomy was performed, and complete resection of the large cystic tumor was successfully achieved, with no postoperative complications. Although a solid pseudopapillary epithelial neoplasm (SPEN) was suspected, specimen morphology, including immunohistochemistry, supported the diagnosis of an adult benign pancreatic hemangioma.

**Conclusion:**

Although mucinous cystic neoplasm (MCN) and adenocarcinoma are the most common pancreatic tumors during pregnancy, various other malignant and benign lesions can be encountered. This report adds to the very small number of pancreatic hemangiomas reported in the literature and involves the first patient diagnosed with this rare condition during pregnancy. Careful clinical considerations regarding diagnostic workup and treatments are required to ensure that mother and child receive the best possible care.

## Background

In pregnant women, the acute abdomen is often a challenge. Careful clinical evaluation, close cooperation between the surgeon and the gynecologist as needed, and the application of appropriate diagnostic tools and educated judgment remain the cornerstones of standard care [1–6]. Although the diagnostic workup can be demanding in these cases, a responsible surgeon might regard appropriate surgical treatment as stressful for the relatively common diseases encountered during pregnancy such as appendicitis [7, 8] and acute cholecystitis [9, 10]. A surgeon is confronted by even greater responsibility for a mother and her child in clinical settings involving rare acute abdominal conditions of pregnancy, which include acute severe pancreatitis [11], gastrointestinal bleeding [12, 13], and suspected tumors in various locations [14–19]. In cases involving a cystic lesion, the nature of the lesion, including its malignant potential and the risk of a spontaneous rupture, must be considered when discussing indications for and the timing of surgery [15, 20]. In this situation, the surgical approach, which varies depending on the pregnancy trimester is a particularly concerning issue.

In this report, we discuss clinical considerations related to the unexpected finding of a large cystic lesion that appears to be related to the pancreas in a pregnant woman with acute abdomen.

### Ethics

Written informed consent was obtained from the patient for publication of this Case report and any accompanying images. A copy of the written consent is available for review by the Editor of this journal.

## Case presentation

**Fig. 1:**
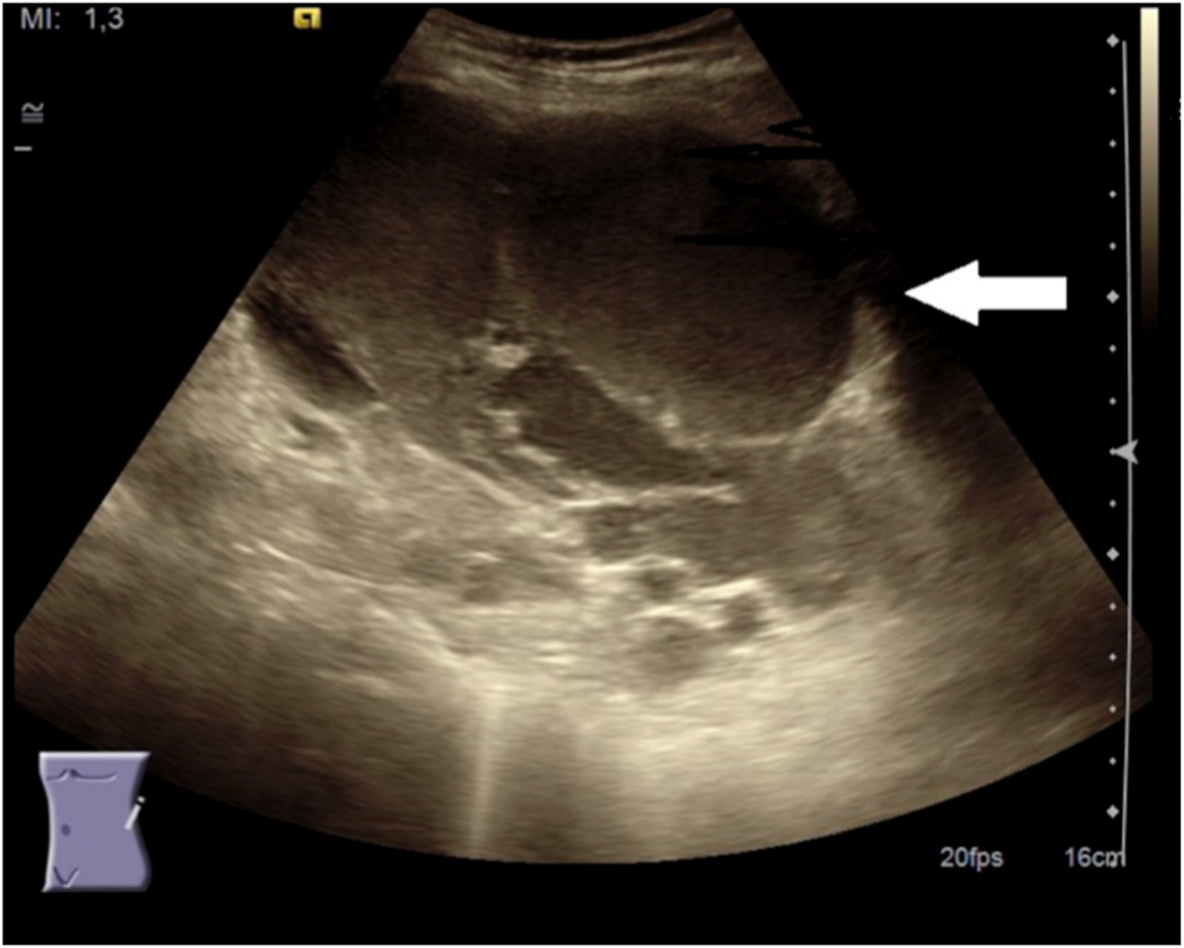
Ultrasound showed a large cystic lesion with septa and some sedimentation. Minimal Doppler signal of the large tumor (18 × 10 cm). Spleen, pancreas and left kidney without focal lesions

**Fig. 2:**
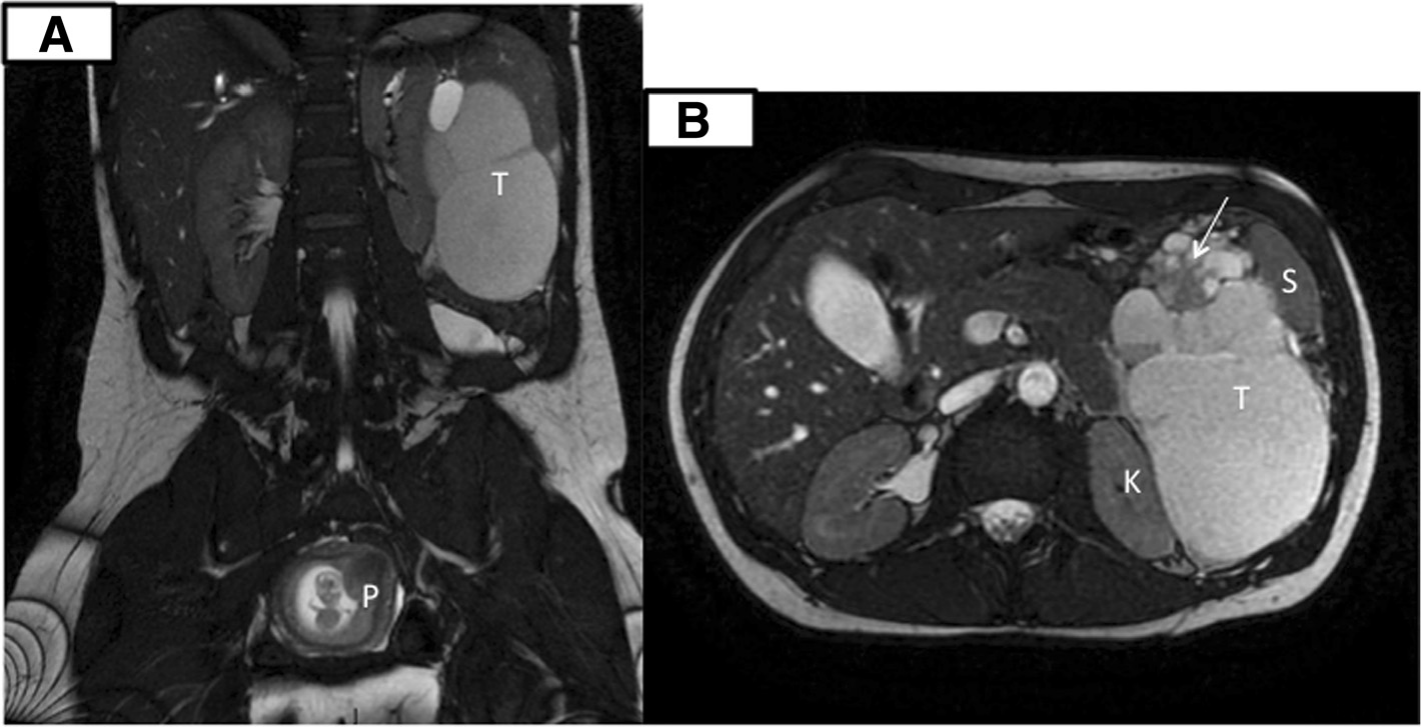
Coronal (**a**) and transversal (**b**) MRI T2 (FIESTA) view. Large tumor (T) medial to the spleen (S) and adjacent to the pancreatic tail dislocating the left kidney (K) medial and cranial. Mostly high signal intensity at T2 and intermediate to high at T1, indicating cystic components with protein or blood. A smaller part of the lesion had lower signal and diffusion restriction indicating more solid parts. (P) is the pregnant uterus. A solid pseudopapillary epithel neoplasia (SPEN) of the pancreas was suspected in this young woman with an encapsulated lesion with cystic, solid and hemorrhagic components

An otherwise healthy, non-obese 30-year-old woman, para 0, at 12 weeks gestation was admitted to our hospital’s Department of Gastrointestinal Surgery with a history of 3 weeks of varying but increasing pain in the left upper quadrant of her abdomen and nausea without vomiting. A palpable left subcostal mass was detected by clinical examination, which revealed no other notable findings except for her pregnant status. Ultrasound (US) examination (Fig. 1) revealed a large cystic lesion (size, 17 × 10 cm) with septa related to the tail of the pancreas and spleen. Magnetic resonance imaging (MRI) depicted a multicystic lesion related to the pancreatic tail, between the spleen and left kidney, with moderate dislocation of both the kidney and spleen (Fig. 2). Routine biochemistry was normal, and tumor markers carcinoembryomic antigen (CEA = 4 μg/l), cancer antigen 125 (CA125 = 42kU/l), carbohydrate antigen 19–9 (CA19–9 < 5 kU/l), and chromogranin A(CgA = 2.8 nmol/l) were all within normal ranges. The preoperative diagnosis was uncertain; however, based on imaging findings and the patient’s young age, a solid pseudopapillary epithelial neoplasm (SPEN) was considered. A gynecologist evaluated the patient’s pregnancy as normal. Relevant factors when considering indications for surgery included the presence of increasing clinical symptoms in a pregnant woman, the uncertain nature of the rather large cystic pancreatic tumor, and the possible risk and undesirable consequences of a rupture of this tumor during the second or third trimester. In accordance with the patient’s preferences, surgical treatment was recommended. Open resection of the most distal portion of the pancreas, including the large cystic tumor and spleen, was performed. Frozen sections of the pancreas resection border confirmed that the resected tissue was benign. The patient’s postoperative course was uneventful, and she was able to leave the hospital on the 6^th^ postoperative day without complications.

**Fig. 3:**
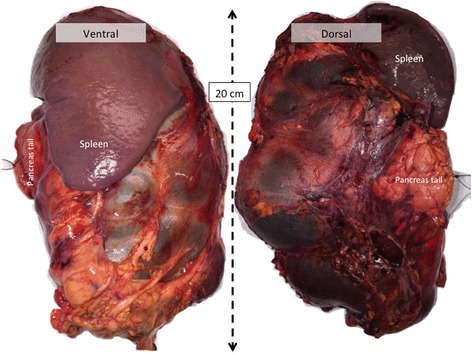
Operative specimen with the cystic tumor and the spleen seen from the ventral (*left*) and the dorsal (*right*) side. The cystic tumor measured 19.5 × 10 × 7 cm

**Fig. 4:**
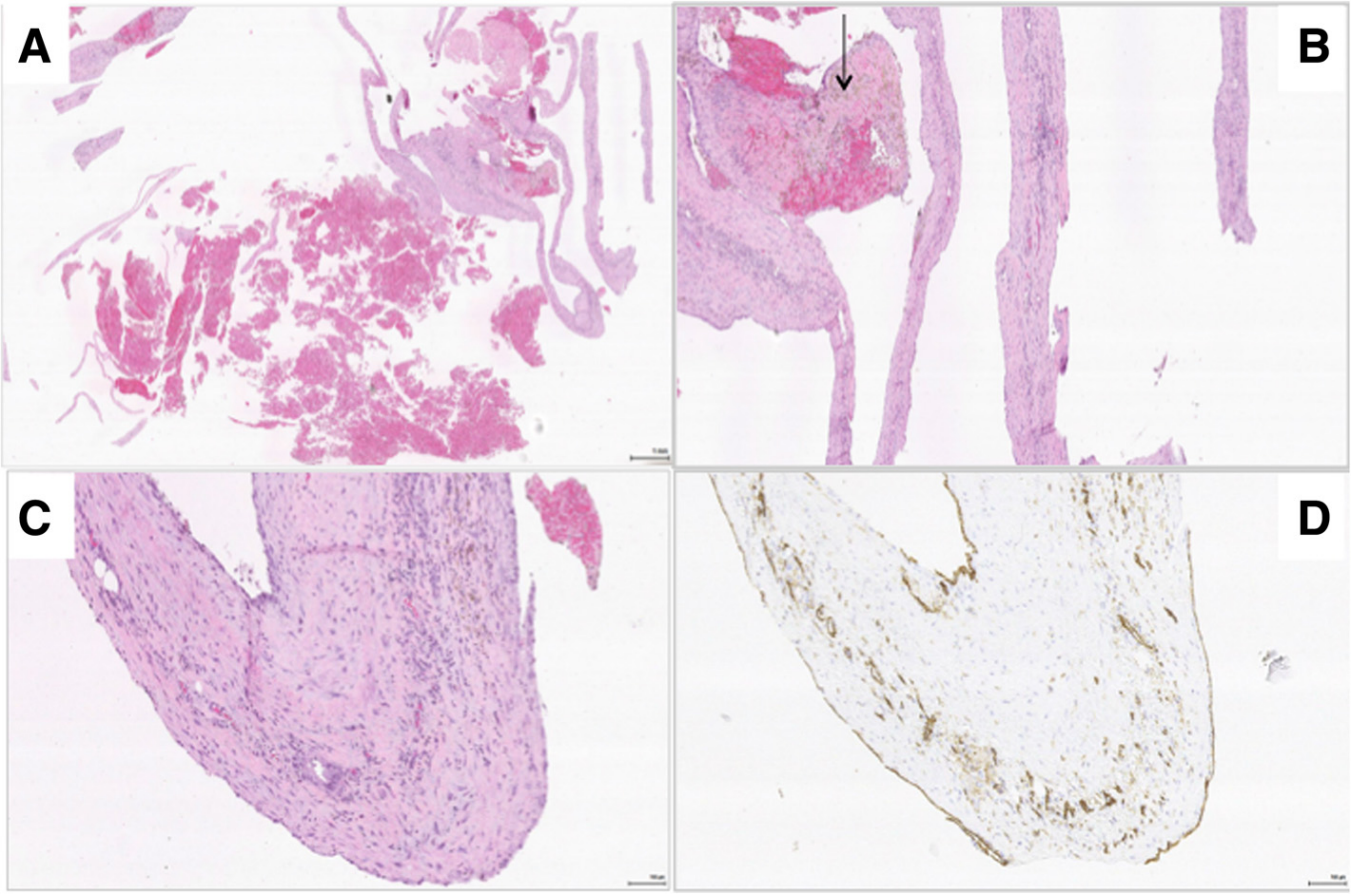
**a** Large dilated vascular structures lined by endothelial cells filled with red blood cells. The thickened vessel walls are composed of fibrous or spindle cell stroma with mild chronic inflammation. (H and E; original magnification: ×10). **b** Large dilated vascular structures lined by endothelial cells filled with red blood cells. The thickened vessel walls are composed of fibrous or spindle cell stroma with mild chronic inflammation and hemosiderin loaded macrophages (arrow) (H and E; original magnification: ×20). **c** Microscopy showing large dilated vascular structure lined by endothelial cells. The wall is infiltrated with mild chronic inflammation. (H and E; original magnification: ×100). **d**. Immunohistochemical expression of CD31 (brown) depicting the endothelial lining of larger vascular spaces and small vessels in the stroma (original magnification, ×20)

The multicystic tumor (Fig. 3) measured 19.5 × 10 × 7 cm and exhibited close adherence to the distal pancreas but without infiltration in the pancreas parenchyma or any communication with the pancreatic ducts. Microscopically, benign pancreatic tissue was confirmed, and the tumor was multicystic with extremely thin septa, fibrosis and a pattern of chronic inflammation, but no epithelial tissue indicating that SPEN was unlikely. Additional immunohistochemistry (IHC) with CD34 and CD 31 (Fig. 4) provided information to support a large-vessel hemangioma lined with single endothelial layer without cytologic atypia, and with focal degenerative changes in septa and the cystic wall. This endothelial lining was negative for cytokeratins and calretinin, excluding epitehelial and mestothelial nature of the lesion Taken together, the tumor morphological findings were characteristic of a truly benign lesion most consistent with adult pancreatic hemangioma, which was resected completely without any ruptures.

After surgery, the patient’s pregnancy proceeded uneventfully, and she spontaneously delivered a healthy child at the calculated gestational time. During follow-up of at least 12 postoperative months, the patient has expressed no complaints or concerns related to her treatment.

## Discussion

While hemangiomas are rarely found in the pancreas, they are very common in the liver. Mundinger et al. reviewed the literature and found only 9 cases with confirmed adult pancreatic hemangiomas reported between 1939 and 2009 [21]. The lesions were most commonly located in the head, or head/body of the gland, with a size varying from 3 cm to 20 cm in diameter. Recently, Bursics and coworkers [22] reported on another patient (a 72-year-old man) surgically treated for a confirmed adult hemangioma of the pancreas, and presented relevant data on the 12 reported cases, including their own, published until 2013. Male:female ratio was found to be 2:1, and the median age was 61 (range, 30–79) years. As demonstrated by these 12 reported cases in the world literature [21, 22], pain was the most common clinical symptom which was also the case in our patient. The pattern of a suggested cystic lesion by imaging was found in most patients with available information in this regard. Notably, the CT patterns of a pancreatic hemangioma are different from the CT signs of a liver hemangioma, as a typical early peripheral contrast-enhancement during the arterial phase is missing in the former [23]. Therefore, this imaging modality is ineffective for ruling out pancreatic hemangioma.

Our patient was definitely in the younger range of age, and importantly, we report on the first pregnant patient diagnosed with an adult pancreas hemangioma. Although the final morphologic diagnosis of this rare condition can be challenging, CD 31 and CD 34 immunohistochemical labeling adds valuable support to the diagnosis of a neoplasm of vascular origin [21].

Both benign and malignant pancreatic neoplasms are rarely diagnosed during pregnancy; in particular, pancreatic cancer during pregnancy is extremely rare, with fewer than 10 described cases to date [15, 16]. However, a number of dilemmas can be encountered when attempting to determine an accurate diagnosis to enable appropriate treatment. Within this context, both US and MRI are reliable and useful imaging modalities [5, 20] without known health risks for the fetus [24]. When indicated, CT can also contribute to imaging findings. Of note, the risk that the fetus will develop congenital abnormalities due to the side effects of radiation exposure (via repeat and/or multiphase CT scans) is considered to be extremely low [25], nonetheless, due to concerns regarding the potential for deleterious side effects, radiation and various contrast agents should be limited and used with care [24, 25].

Del Chiaro et al. [26] recently demonstrated that the overall accuracy of preoperative diagnoses of cystic pancreatic lesions is only approximately 60 %, with similar accuracies for asymptomatic and symptomatic lesions. Thus, inaccurate preoperative assessments of pancreatic cystic lesions are common; however, diagnostic errors are clinically relevant in less than 10 % of these cases. Data regarding lesion size, patient gender, and the patient’s prior history (with respect to pancreatitis or other relevant diseases) could be valuable information for reaching appropriate decisions and thereby preventing the overutilization of operative resection in patients with these lesions [27]. For non-pregnant patients, additional imaging (e.g., contrast CT scans) and endoscopic ultrasound (EUS)-guided aspiration of the cystic lesion for analyses of DNA mutations and proteins within pancreatic cyst fluid can contribute to the diagnostic workup [20, 28]. However, due to potential risks for a biopsy related bleeding, rupture of the lesion or peritoneal seeding of biopsy material, we did not regard our symptomatic pregnant patient with a large pancreatic lesion as a candidate for this diagnostic approach. Her subjective and increasing complaints, the uncertain nature of the lesion, and the risk of tumor rupture with undesired side effects or complications later during her pregnancy, were all important considerations for the timing of treatment. The preoperative diagnosis of a possible SPEN was not definitively determined. As recently suggested [29] SPENs are rare cystic lesions that frequently occur in young women and patients with these lesions exhibit good prognoses if radical surgical resection can be achieved. Despite the challenging nature of a definite diagnosis, the only feature of the described case that could be associated with malignancy was a large tumor size at diagnosis [20, 29]. Thus fare, malignancy has not been reported for pancreatic hemangiomas. Based on the rarity of this condition, the lack of reliable follow-up data, and the sparse literature on this topic, an understanding of the pathophysiology and natural history of these lesions remains at an early stage [21].

## Conclusion

Although reports have described a number of anecdotes involving worrisome histories of pregnant women with malignant tumors, including carcinomas of the pancreas [16, 30, 31], pancreatic neuroendocrine tumors [14], and bleeding neoplasms [32], or spontaneous SPEN rupture [33], these cases are generally exceptions rather than the rule. Nevertheless, given the objective of providing optimal care to both mother and child, the determination of an appropriate treatment schedule for any young pregnant woman requires the consideration of a wide range of diagnoses and a number of important factors. Although the second trimester is considered to be the most favorable time for surgical intervention for pancreatic tumors [15], multidisciplinary consultation should occur with respect to a pregnant patient’s diagnosis, indications and timing to ensure that the best possible outcomes for both the mother and the child are achieved [15, 18, 19, 22, 26].

## Abbreviations

CT: Computer tomography
EUS: Endoscopic ultrasound
IHC: Immunohistochemistry
MCN: Mucinous cystic neoplasm
MRI: Magnetic resonance imaging
SPEN: Solid pseudopapillary epithelial neoplasm
